# Clinical course and factors associated with progressive acro-osteolysis in early systemic sclerosis: a retrospective cohort study

**DOI:** 10.1038/s41598-024-55877-x

**Published:** 2024-03-01

**Authors:** Punthip Thammaroj, Prathana Chowchuen, Chingching Foocharoen

**Affiliations:** 1 Department of Radiology, Faculty of Medicine, Khon Kaen University, 123 Mittraphap Rd, Nai-Mueang, Mueang District, Khon Kaen, 40002 Thailand; 2 Division of Rheumatology, Department of Medicine, Faculty of Medicine, Khon Kaen University, Khon Kaen, Thailand

**Keywords:** Systemic sclerosis, Scleroderma and related disorders, Acro-osteolysis, Hand radiography, Cohort study, Diseases, Rheumatology, Risk factors, Signs and symptoms

## Abstract

To examine clinical course of early systemic sclerosis (SSc) and identify factors for progression of acro-osteolysis by a retrospective cohort study. Dual time-point hand radiography was performed at median interval (range 3.0 ± 0.4 years) in 64 recruited patients. Progressive acro-osteolysis was defined as the worsening of severity of acro-osteolysis according to rating scale (normal, mild, moderate, and severe). Incidence of the progression was determined. Cox regression was analyzed for the predictors**.** A total of 193.6 per 100 person-years, 19/64 patients had progressive acro-osteolysis with incidence of 9.8 per 100-person-years (95% CI 6.3–15.4). The median time of progressive acro-osteolysis was 3.5 years. Rate of progression increased from 1st to 3rd years follow-up with the progression rate at 1-, 2- and 3-years were 0, 2.0 and 18.3%, respectively. Patients with positive anti-topoisomerase I tended to have more progressive acro-osteolysis but no significant predictors on Cox regression. 44%, 18%, and 33% of who had no, mild, and moderate acro-osteolysis previously developed progression and 10 turned to be severe acro-osteolysis. In conclusion, the incidence of progressive acro-osteolysis was uncommon in early SSc but the rate of progression was pronouncedly increasing after three years follow-up. A half of the patients progressed to severe acro-osteolysis.

## Introduction

Systemic sclerosis (SSc) is an uncommon connective tissue disease of unknown etiology characterized by vascular, immune and fibrotic changes in the skin and some internal organs. The prognosis varies depending on the extent of the skin compromise, the degree of involvement of internal organs and the comorbidities^1,2^.

Acro-osteolysis, an erosion of distal phalanges, is a manifestation of musculoskeletal involvement in SSc. The prevalence of acro-osteolysis of the hands was reported around 64% of the early SSc patients with the range of 20–76% depending on the study population^2,3^. The process of acro-osteolysis usually starts at the tip of fingers and progresses towards the destruction of a large portion of the distal phalanx, giving the finger a conical appearance in the most severe cases. Its clinical importance, is its association with digital ulcers, severe Raynaud’s phenomenon, calcinosis, and MDSS (The Medsger severity scale). Furthermore, associated with anti-Scl70 and hand deformity in patients with early SSc^2^.

Although the clinical importance of acro-osteolysis has been reported, there is no recently longitudinal data, precluding the identification of predictors of the progression of acro-osteolysis in patients with early SSc. We therefore examined the course of the acro-osteolysis, to define the incidence, and to identify the risk factor to predict the progression of acro-osteolysis in the patients with early SSc.

## Methods

This was a longitudinal retrospective cohort study of hand radiographs and clinical features of the SSc patients attended the Scleroderma Clinic, Khon Kaen University Hospital, Thailand from April 2020 to March 2023.

### Study population

We considered eligible any SSc patients over 18 years of age and (a) met 2013 American College of Rheumatology (ACR)/European Alliance of Associations for Rheumatology (EULAR) Classification Criteria for SSc^4^, (b) had a time interval of less than 4 years from the date of onset of SSc to the date of the first hand radiographic examination, which was defined as an early SSc, and, (c) underwent hand radiography at least twice at least 2 years apart. The study period was from April 2020 to March 2023. The patients diagnosed as having an overlap syndrome were excluded.

### Clinical variables

We carried out a general evaluation of these patients twice, the first evaluation at the time of the first hand radiographic examination and then the second hand radiographic examination. The data included age, sex, subsets of SSc (diffuse cutaneous SSc (dcSSc) or limited cutaneous SSc (lcSSc)), presence of anti-topoisomerase I antibody (anti-topo I), SSc clinical characteristics, and the modified Rodnan skin score (mRSS).

### Hand radiographs

All eligible patients underwent hand radiographs in 2 positions; posteroanterior and oblique views using a general radiography system (Hitachi, Radnext 80, 50 kVp, 1 mAs) at the first and the second evaluation time-point.

### Operation definitions

Diagnosis of SSc was based on the 2013 ACR/EULAR Classification Criteria for SSc^4^. The SSc patients were classified into dcSSc and lcSSc groups according to LeRoy et al. 1988^5^. The onset of SSc was defined as the time of the first development of the first non-Raynaud SSc symptom reported by the patient. Pulmonary arterial hypertension (PAH) is defined as the mean pulmonary arterial pressure > 20 mmHg confirmed by right heart catheterization^6^. The presence of interstitial lung disease (ILD) was considered when interstitial fibrosis was detected by high-resolution computed tomography (HRCT). Edematous skin was characterized as the feature of puffy hand or non-pitting oedema of fingers. Hand deformity was defined when flexion contractures of the finger joints.

### Analysis of radiographs

All hand radiographs were filed and reviewed using the Picture Archive and Communication Systems (PACS). Radiographic examination for acro-osteolysis was performed by two 10-years-experienced musculoskeletal radiologists. Any discrepancy was given in a consensus.

**Figure 1:**
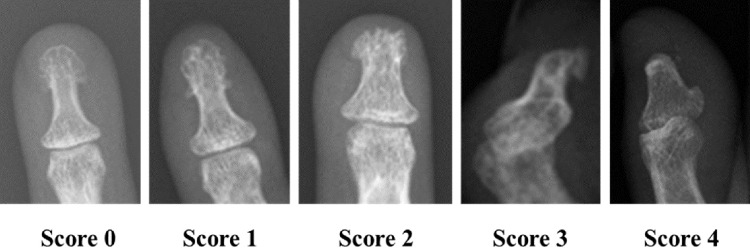
Scoring of acro-osteolysis. Score 0: normal terminal phalanges, no resorption; Score 1: minimal acro-osteolysis, small amount of resorption at the terminal tuft; Score 2: resorption of most of the distal tip of the terminal tuft; Score 3: resorption of most of the terminal tuft, leaving only one side intact; Score 4: complete resorption of the terminal tuft, with obvious penciling.

Acro-osteolysis was defined as the bone resorption at the terminal tuft of the fingers. A grading scale for the severity of acro-osteolysis was used referring to the scale from 0 to 4 that took into account the presence and severity of acro-osteolysis of each finger (including thumbs), with 0 being normal bone structure and 4 being severe penciling of the terminal phalanx^3,7,8^ (Fig. 1).

The grading scale is as follows:

0. Normal terminal phalanges. No resorption.

1. Minimal acro-osteolysis, small amount of resorption at the terminal tuft.

2. Resorption of most of the distal tip of the terminal tuft.

3. Resorption of most of the terminal tuft, leaving only one side intact.

4. Complete resorption of the terminal tuft, with obvious penciling.

The overall rating scale consisting of normal, mild, moderate or severe acro-osteolysis was described to each patient based on the following criteria:

0. Normal.

1. Mild acro-osteolysis: the maximum score for an individual finger = 1 and the total score (the sum of the scores of all fingers) ≤ 8.

2. Moderate acro-osteolysis: the maximum score for an individual finger ≤ 2 and the total score ranging from 9 to 16.

3. Severe acro-osteolysis: the maximum score for an individual finger ≥ 3 or the total score ≥ 17.

### Criteria for SSc with and without progressive acro-osteolysis

Progressive acro-osteolysis was fulfilled when there was worsening of acro-osteolysis severity by the overall rating scale of an individual patient at the 2nd radiographic evaluation compared to that at the 1st radiographic evaluation; for instance, from mild to moderate, moderate to severe acro-osteolysis. In other words, progressive acro-osteolysis is defined as the increase of both the maximum score for an individual finger and the total score (sum of all fingers) at the 2nd radiographic examination compared to that at the 1st radiographic evaluation.

### Statistical analysis

Continuous variables were presented as mean (± standard deviation) or median (± interquartile range [IQR]), as appropriate. Categorical variables were given as number and percentage. Incidence rate of progressive acro-osteolysis and its 95% confidence interval (95% CI) were evaluated. Kaplan–Meier was used to estimate the probability of the progressive acro-osteolysis and the median time to develop progressive acro-osteolysis. Cox regression was used to assess the factors associated with progressive acro-osteolysis. The hazard ratio and the 95% CI between prognosis factors and the progression of acro-osteolysis were assessed.

All statistical tests were two-tailed. A *P* value of < 0.05 was considered statistically significant. All of the data analyses were performed using STATA version 16.0 (StatCorp., College Station, TX, US).

### Ethical approval

This study was designed by the authors and approved by the Human Research Ethics Committee of Khon Kaen University with the approval number HE651109 as per the Helsinki Declaration and the Good Clinical Practice Guidelines. All eligible patients signed informed consent before enrolment.

## Results

### Evaluation of clinical features

A total of 64 SSc patients were included, of whom 39 (60.9%) patients were females and 47 of 62 (75.8%) were dcSSc. The mean age at the time of the 1st radiographic evaluation and that at the time of the 2nd radiographic evaluation was 57.1 ± 9.7 and 60.0 ± 9.4 years, respectively. The mean disease duration at the time of the 2nd radiographic evaluation was 4.4 ± 1.2 years. The mean interval between the 1st and the 2nd radiographic evaluation was 3.0 ± 0.4 years. Anti-topo I antibody (anti-topo I) data were available for 54 patients and the majority of patients (45/54, 83.3%) were positive. While almost all of patients with progressive acro-osteolysis were positive to anti-topo I, the anti-topo I positive rate of those without progressive acro-osteolysis were nearly 80% (32/41). The overall clinical characteristics of SSc patients with and without progressive acro-osteolysis patients were shown in Table 1.

**Table 1:** Predictors of progressive acro-osteolysis by univariate analysis.

Data	Overall N = 64	Without progressive acro-osteolysis N = 45	With Progressive acro-osteolysis N = 19	HR (95% CI)	p value
Female (%)	39 (60.9)	30 (66.7)	9 (47.4)	0.53 (0.21–1.31)	0.17
Diffuse cutaneous systemic sclerosis (%)	47 of 62 (75.8)	32 (74.4)	15 (79.0)	0.98 (0.32–2.96)	0.97
Age at the 1st radiographic evaluation; years (mean ± SD)	57.1 ± 9.7	56.8 ± 9.3	57.7 ± 10.7	1.01 (0.96–1.07)	0.61
Age at the 2nd radiographic evaluation; years (mean ± SD)	60.0 ± 9.4	59.8 ± 9.1	60.3 ± 10.2	1.01 (0.96–1.07)	0.57
Duration of disease at 2nd radiographic evaluation; years (mean ± SD)	4.4 ± 1.2	4.2 ± 1.0	4.7 ± 1.4	1.15 (0.74–1.77)	0.54
Interval between the 1^st^ and the 2nd radiographic evaluation; years (mean ± SD)	3.0 ± 0.4	3.1 ± 0.4	2.9 ± 0.4	NA	NA
Anti-topoisomerase I antibody positive (%)	45 of 54 (83.3)	32 (78.1)	13 (100)	NA	NA
Clinical features at the 1st radiographic evaluation					
Raynaud’s phenomenon (%)	18 of 60 (30.0)	14 (32.6)	4 (23.5)	0.62 (0.20–1.90)	0.40
Ischemic ulcer (%)	7 of 60 (11.7)	5 (11.6)	2 (11.8)	0.89 (0.20–3.92)	0.88
Digital gangrene (%)	1 of 60 (1.7)	1 (2.3)	0 (0.0)	NA	NA
Telangiectasia (%)	14 of 60 (23.3)	10 (23.3)	4 (23.5)	0.88 (0.28–2.74)	0.83
Salt and pepper skin (%)	33 of 60 (55.0)	23 (53.5)	10 (58.8)	1.02 (0.39–2.68)	0.99
Edematous skin (%)	9 of 60 (15.0)	7 (16.3)	2 (11.8)	0.67 (0.15–2.96)	0.60
Tendon friction rub (%)	8 of 60 (13.3)	5 (11.6)	3 (17.7)	1.46 (0.42–5.10)	0.55
Hand deformities (%)	23 of 60 (38.3)	17 (39.5)	16 (35.3)	1.18 (0.43–3.24)	0.75
Synovitis (%)	1 of 60 (1.7)	1 (2.3)	0 (0.0)	NA	NA
mRSS; points (median (IQR))	8.0 (2.0–13.0)	9.0 (2.0–15.5)	2.0 (2.0–10.0)	0.97 (0.92–1.03)	0.35
Pulmonary fibrosis (%)	17 of 58 (29.3)	11 (26.2)	6 (37.5)	1.57 (0.57–4.36)	0.38
Pulmonary hypertension (%)	3 of 58 (5.2)	2 (4.8)	1 (6.3)	1.92 (0.25–14.95)	0.53
Renal crisis (%)	0 of 58 (0.0)	0 (0.0)	0 (0.0)	NA	NA
Clinical features at the 2nd radiographic evaluation					
Raynaud’s phenomenon (%)	27 of 62 (43.6)	17 (39.5)	10 (52.6)	1.40 (0.57–3.45)	0.47
Ischemic ulcer (%)	7 of 62 (11.3)	5 (11.6)	2 (10.5)	0.93 (0.21–4.05)	0.92
Digital gangrene (%)	0 of 62 (0.0)	0 (0.0)	0 (0.0)	NA	NA
Telangiectasia (%)	26 of 62 (41.9)	19 (44.2)	7 (36.8)	0.58 (0.23–1.49)	0.26
Salt and pepper skin (%)	29 of 62 (46.8)	19 (44.2)	10 (52.6)	1.29 (0.52–3.19)	0.58
Edematous skin (%)	2 of 62 (3.2)	2 (4.7)	0 (0.0)	NA	NA
Tendon friction rub (%)	8 of 62 (12.9)	2 (14.0)	3 (10.6)	0.74 (0.17–3.19)	0.68
Hand deformities (%)	20 of 62 (32.3)	14 (32.6)	6 (31.6)	0.80 (0.30–2.12)	0.66
Synovitis (%)	1 of 62 (1.6)	1 (2.3)	0 (0.0)	NA	NA
mRSS; points (median (IQR))	2.0 (0.0–9.0)	2 (0.0–8.0)	4(0.0–10.0)	1.02 (0.94–1.09)	0.70
Pulmonary fibrosis (%)	32 of 62 (51.6)	24 (55.8)	8 (42.1)	0.70 (0.28–1.74)	0.44
Pulmonary hypertension (%)	6 of 61 (9.8)	5 (11.9)	1 (5.3)	0.75 (0.10–5.70)	0.78
Renal crisis (%)	0 of 62 (0.0)	0 (0.0)	0 (0.0)	NA	NA

*mRSS *modified Rodnan skin score, *ESR *erythrocyte sedimentation rate, *CRP *C-reactive protein, * statistical significant, *SD *standard deviation, *IQR *interquartile range, *NA *data not available due to statistical limitation*.*

### Progression rate of acro-osteolysis in patients with SSc

**Figure 2:**
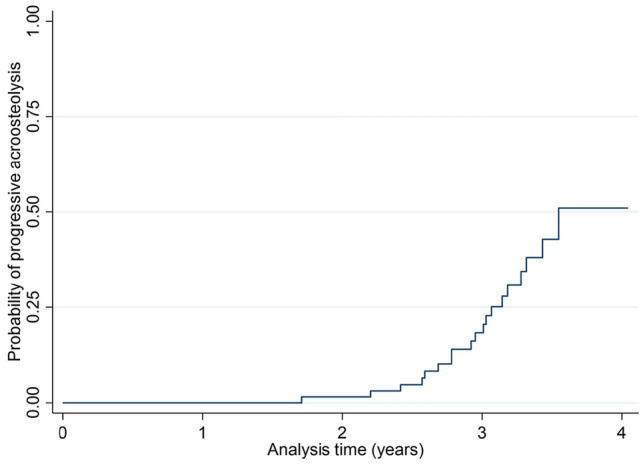
Cumulative incidence of progression of acro-osteolysis in patients with SSc.

Among a total of 64 SSc patients with the incidence of 193.6 per 100 person-years, 19 cases were defined as having progressive acro-osteolysis with the incidence of 9.8 per 100-person-years (95% CI 6.3–15.4). The median time of the development of progressive acro-osteolysis was 3.5 years. The progression rate at 1-, 2-, and 3-years was 0.0, 2.0, and 18.3%, respectively. The cumulative incidence of progression acro-osteolysis in patients with SSc is presented in Fig. 2.

### Changing patterns of progressive acro-osteolysis using an overall rating scale

**Figure 3:**
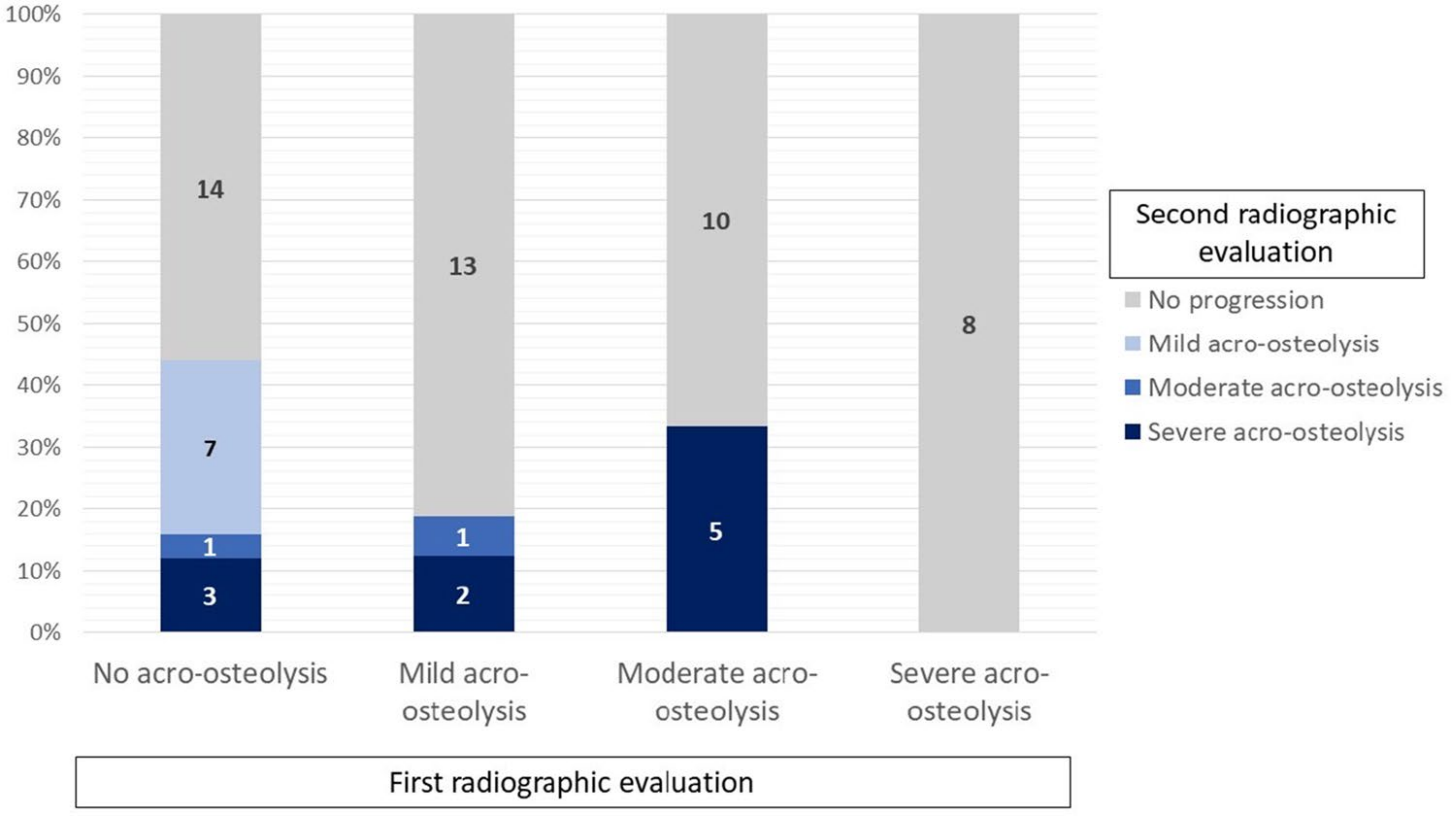
Changing patterns of the severity of progressive acro-osteolysis.

At the 1st radiographic evaluation, 25 of 64 early SSc patients were identified not to have acro-osteolysis, 16 were mild acro-osteolysis, 15 were moderate, and 8 were severe acro-osteolysis. During the follow-up period, 11 (44%) of 25 cases of SSc patients without acro-osteolysis developed acro-osteolysis; 7 cases (36.8%) developed mild acro-osteolysis, 1 case moderate, and 3 cases developed severe acro-osteolysis. Among 16 cases with mild acro-osteolysis previously, 1 case progressed to moderate and 2 cases to severe acro-osteolysis. and around 33% of who had moderate acro-osteolysis previously had progressive acro-osteolysis by the 2nd radiographic evaluation (Fig. 3).

### Predictors of progressive acro-osteolysis

Among various clinical parameters related to SSc, male patients, the presence of anti-topo I, and having Raynaud’s phenomenon on the 2nd radiographic evaluation were more frequently observed among patients with progressive acro-osteolysis than those without progressive acro-osteolysis, although the differences were statistically not significant by univariate analysis (Table 1). The presence of anti-topo I tended to be a predictor of progressive acro-osteolysis.

When the worsening of the severity of acro-osteolysis at least one finger of individual patient at the 2nd radiographic evaluation compared to the 1st radiographic evaluation was used as the criterion of progressive acro-osteolysis, 28 (43.8%) out of 64 SSc patients were classified to have progressive acro-osteolysis. By this classification, clinical features of SSc patients with and without progressive acro-osteolysis were compared. The findings were given in Supplementary Table 1. Apparently the results were essentially the same as those seen in the evaluation of the clinical features of SSc patients with/without progressive acro-osteolysis defined by the worsening of the severity based the overall rating scale (Table 1). Again, presence of anti-topo I antibody can be a predictor of progressive acro-osteolysis.

## Discussion

Our study is the first to identify clinical predictors of progressive acro-osteolysis in an early SSc using systematic examination and scoring systems of the dual time point of radiographic evaluation. The incidence of progressive acro-osteolysis was rather low, about 9.8 per 100-person-years. The median time of the development of progressive acro-osteolysis was 3.5 years. During follow-up, progression from no acro-osteolysis to mild acro-osteolysis is most commonly observed, and a half of the patients showed progression to severe acro-osteolysis.

In terms of the progression rate of acro-osteolysis, we found that within the first 2 years of the follow-up of the patients with early SSc, not many patients have progressive acro-osteolysis. However, the later stage of 3-year-follow-up, the incidence of progression increased. According to the study methodology, radiographic study was followed up 2 years after the initial study, thus lacking data on acro-osteolysis changes within a shorter follow-up duration. While radiographic progression might have occurred during the interval between the 1st and 2nd radiographic study, the precise starting of the progression remains unknown. Nevertheless, we recommend prompt intervention targeting the prevention of progression within 3 years after the 1st radiographic evaluation before acro-osteolysis becomes apparent, in particular, there is currently no intervention that can prevent acro-osteolysis. Additionally, targeting vasculopathy and vitamin D supplementation in case of vitamin D deficiency should be considered to mitigate the condition^9^. Hand radiograph follow-up should be also performed within the 3 years to monitor changes and assess the severity of acro-osteolysis.

A proportion of SSc patients showed rapid progression of acro-osteolysis. We found that 6 of our cases showed worsening of the severity more than 1 level (1 from normal to moderate, 3 from normal to severe, and 2 from mild to severe acro-osteolysis). Unfortunately, in this study, we were unable to find any significant clinical predictors for the progressive acro-osteolysis. This might be due to low power of statistical method that related to the low numbers of the event (progressive acro-osteolysis) and a small sample size. We are now planning to conduct long-term follow-up on those who had progressive acro-osteolysis for the better understanding of the nature of the disease and its long-term outcomes.

Although there were no clinical characteristics as the predictor for progressive acro-osteolysis in our study, we found that male SSc and the presence of anti-topo I antibody at the 2nd radiographic evaluation were more frequent in the patients with progressive acro-osteolysis than in those without progression. The findings might be explained by the worse prognosis among male SSc and who were positive for anti-topo I. Anti-topo I is one of the antibodies in SSc, which appears to be more frequently in patients with not only internal organ involvement (i.e., ILD) but also digital tip ulcers and digital tuft resorption^3,10–12^. Based on our findings, progression of acro-osteolysis should be aware in an early SSc patients, particularly in male SSc and who has positive for anti-topo I.

As reported previously by others^8,9,13^, acro-osteolysis resulted associated with digital ulcers. We did not find any statistical significance between progressive acro-osteolysis with clinical of digital ulcers. This might be explained by the low prevalence of vasculopathy, ischemic ulcer, and digital gangrene among Thais SSc related to the warm/hot climate of Thailand. Raynaud’ phenomenon might be unobvious to detect and easily ignored^14,15^.

Although statistically not significant, Raynaud’s phenomenon is more commonly seen in the patients with progressive acro-osteolysis than in those without progression. According to the pathophysiology of Raynaud’s phenomenon, it is a painful vascular condition in which abnormal vasoconstriction of the digital arteries and is aggravated by cold exposure. This can be presented in an early disease, associated with digital ulcers, and consequently related to acro-osteolysis. The principle of the treatment for Raynaud’s phenomenon is vasodilator therapy such as calcium channel blockers to reduce critical ischemia in the digits^16–18^. We hypothesize that if vasodilators were given at an adequate dose and appropriate time, it might delay the progression of acro-osteolysis by the time. This possibility should be further investigated.

Currently, there is no standardized classification, neither an overall rating scale nor even a single finger-progression to define patients experiencing progression or lack thereof in acro-osteolysis. Despite comparable results, we preferred using the overall rating scale as classification method. Our rationale is in the belief that SSc manifests as a systemic disease, making a single-digit change too sensitive for determining further management. Moreover, an overall rating scale might prove more practical and simpler for patient follow-up, categorizing degree changes as mild, moderate, or severe acro-osteolysis.

This study has some limitations. First, the number of patients was too small to evaluate accurately the relationships between clinical features and the progressive acro-osteolysis at dual time-point radiographic evaluation. Second, we did not analyze the effect of treatment on the progression of acro-osteolysis because the given treatments were too variable depending on the organ involved. Third, the prevalence of the acro-osteolysis in this study might overestimate due to a selection bias. Forth, because this is a single center study, the findings might not be generalized. Lastly, in terms of the clinical feature analysis, we evaluate synovitis only by clinical examination and some laboratory parameters, but not used ultrasound or magnetic resonance imaging. The strength of our study is; this is a first report of the longitudinal radiographic follow-up of early SSc, so the findings will help us to understand the nature of bone involvement in early SSc and guide use for further intervention planning to prevent the development of progressive acro-osteolysis among early SSc patients.

## Conclusion

The incidence of progressive acro-osteolysis was uncommon in patients with early SSc but the rate of progression was pronouncedly increasing after 3 years follow-up.

### Supplementary Information

Supplementary Table 1.

## Data Availability

Data and materials are available upon request by contact to corresponding author.
