# Supplementary material for: Clinical course and factors associated with progressive acro-osteolysis in early systemic sclerosis: a retrospective cohort study
Source: Sci Rep. 2024 Mar 1;14:5129. doi: 10.1038/s41598-024-55877-x (PMC10907566; doi:10.1038/s41598-024-55877-x)
Supplement: Supplementary file 1 — Supplementary Table 1. [file 41598_2024_55877_MOESM1_ESM.pdf]

SUPPLEMENTARY MATERIALS

Predictors of progression of acro-osteolysis based on changes in the severity of acro-osteolysis by grading scale

Supplementary Table S1. Predictors of progressive acro-osteolysis by univariate analysis

Data	Overall N = 64	Without progressive acro-osteolysis N = 36	With Progressive acro-osteolysis N = 28	HR (95%CI)	p-value
Female (%)	39 (60.9)	23 (63.9)	16 (57.1)	0.78 (0.37-1.66)	0.52
Diffuse cutaneous systemic sclerosis (%)	47 of 62 (75.8)	26 (74.3)	21 (77.8)	0.90 (0.36-2.24)	0.83
Age at the 1st radiographic evaluation; years (mean ±SD)	57.1±9.7	55.5±8.6	59.3±10.8	1.04 (0.99-1.10)	0.08
Age at the 2nd radiographic evaluation; years (mean ±SD)	60.0±9.4	58.4±8.5	61.9 ±10.2	1.04 (0.99-1.10)	0.09
Duration of disease at the 2nd radiographic evaluation; years (mean ±SD)	4.4±1.2	4.4±1.0	4.3±1.3	0.83 (0.56-1.23)	0.35
Interval between the 1st and the 2nd radiographic evaluation; years (mean ±SD)	3.0±0.4	3.1±0.4	2.9±0.4	NA	NA
Anti-topoisomerase I antibody positive (%)	45 of 54 (83.3)	24 (75.0)	21(95.5)	3.92 (0.53-29.24)	0.18
**Clinical features at the 1st radiographic evaluation**					
Raynaud's phenomenon (%)	18 of 60 (30.0)	12 (34.3)	6 (24.0)	0.63 (0.25-1.59)	0.33
Ischemic ulcer (%)	7 of 60 (11.7)	2 (5.7)	5 (20.0)	1.66 (0.62-4.43)	0.31
Digital gangrene (%)	1 of 60 (1.7)	0 (0.0)	1 (4.0)	2.02 (0.27-15.16)	0.49
Telangiectasia (%)	14 of 60 (23.3)	9 (25.7)	5 (20.0)	0.72 (0.27-1.92)	0.51
Salt and pepper skin (%)	33 of 60 (55.0)	17 (48.6)	16 (64.0)	1.25 (0.55-2.82)	0.60
Edematous skin (%)	9 of 60 (15.0)	7 (20.0)	2 (8.0)	0.44 (0.10-1.89)	0.27
Tendon friction rub (%)	8 of 60 (13.3)	4 (11.4)	4 (16.0)	1.29 (0.44-3.76)	0.64
Hand deformities (%)	23 of 60 (38.3)	11 (31.4)	12 (48.0)	2.03 (0.91-4.50)	0.08

Synovitis (%)	1 of 60 (1.7)	1 (2.9)	0 (0.0)	NA	NA
mRSS; points (median (IQR))	8.0 (2.0-13.0)	8.0 (2.0-13.0)	7.0 (2.0-14.5)	1.00 (0.96-1.05)	0.80
Pulmonary fibrosis (%)	17 of 58 (29.3)	10 (29.4)	7 (29.2)	1.07 (0.44-2.58)	0.88
Pulmonary hypertension (%)	3 of 58 (5.2)	2 (5.9)	1 (4.2)	1.28 (0.17-9.64)	0.81
Renal crisis (%)	0 of 58 (0.0)	0 (0.0)	0 (0.0)	NA	NA
**Clinical features at the 2nd radiographic evaluation**					
Raynaud's phenomenon (%)	27 of 62 (43.6)	15 (42.9)	12 (44.4)	0.99 (0.46-2.13)	0.99
Ischemic ulcer (%)	7 of 62 (11.3)	4 (11.4)	3 (11.1)	0.95 (0.28-3.17)	0.93
Digital gangrene (%)	0 of 62 (0.0)	0 (0.0)	0 (0.0)	N/A	N/A
Telangiectasia (%)	26 of 62 (41.9)	16 (45.7)	10 (37.0)	0.59 (0.27-1.29)	0.19
Salt and pepper skin (%)	29 of 62 (46.8)	13 (37.14)	16 (59.3)	1.66 (0.77-3.59)	0.19
Edematous skin (%)	2 of 62 (3.2)	2 (5.7)	0 (0.0)	4.44e-15	1.00
Tendon friction rub (%)	8 of 62 (12.9)	5 (14.3)	3 (11.1)	0.78 (0.23-2.60)	0.69
Hand deformities (%)	20 of 62 (32.3)	10 (28.6)	10 (37.0)	1.01 (0.46-2.21)	0.98
Synovitis (%)	1 of 62 (1.6)	1 (2.9)	0 (0.0)	4.46e-15	1.00
mRSS; points (median (IQR))	2 (0.0-9.0)	2 (0.0-6.0)	6 (0.0-10.0)	1.03 (0.97-1.10)	0.27
Pulmonary fibrosis (%)	32 of 62 (51.6)	20 (57.1)	12 (44.4)	0.78 (0.34-1.67)	0.52
Pulmonary hypertension (%)	6 of 61 (9.8)	3 (8.82)	3 (11.1)	1.64 (0.49-5.57)	0.42
Renal crisis (%)	0 of 62 (0.0)	0 (0.0)	0 (0.0)	NA	NA

*mRSS: modified Rodnan skin score; ESR: erythrocyte sedimentation rate; CRP: C-reactive protein; *: statisticaly significant; SD: standard deviation; IQR: interquartile range; NA: data not available*
